# Complete chloroplast genome sequence and phylogenetic analysis of *Chimonobambusa sichuanensis* (Bambusoideae)

**DOI:** 10.1080/23802359.2021.1884017

**Published:** 2021-03-11

**Authors:** Wanqi Zhao, Binbin Cao, Guangyao Yang, Wengen Zhang, Fen Yu

**Affiliations:** Jiangxi Provincial Key Laboratory for Bamboo Germplasm Resources and Utilization, Forestry College, Jiangxi Agricultural University, Nanchang, PR China

**Keywords:** Poaceae, *Chimonobambusa sichuanensis*, phylogeny, chloroplast genome

## Abstract

*Chimonobambusa sichuanensis* is an ornamental shrubby bamboo endemic to southern China. In this study, the complete chloroplast genome (cpDNA) sequence of *Chimonobambusa sichuanensis* was first reported. The cpDNA is 139,594 bp in length, including a small single-copy (SSC) region of 12,820 bp and a large single-copy (LSC) region of 83,196 bp, which were separated by a pair of inverted repeat (IR) regions of 21,789 bp. The genome contains 140 genes, consisting of 93 protein-coding genes, seven ribosomal RNA (rRNA) genes, and 40 transfer RNA (tRNA) genes. The phylogenetic analysis showed that *C. sichuanensis* is highly clustered in the *Phyllostachys* clade, sister to *C. tumidissinoda*.

*Chimonobambusa sichuanensis* (T. P. Yi) T. H. Wen, belonging to the family Poaceae (Bambusoideae: Arundinarieae), is regarded as a representative ornamental bamboo species for its graceful appearance and strong adaptability. The clear taxonomic status of this species is important to understanding the evolution of the genus *Gelidocalamus*. However, the classification of *C. sichuanensis* remains controversial (Yi 1982, 1992). The chloroplast genome sequence has been used to determine evolutionary relationships in many bamboo species, such as *Dendrocalamopsis vario-striata* (Lin et al. 2019), *Gelidocalamus xunwuensis* (Zhang et al. 2019), *Bambusa tulda* (Liu and Lan 2020), *Acidosasa gigantea* (Zheng et al. 2020), *Phyllostachys edulis* ‘Pachyloen’ (Huang et al. 2019) and *Phyllostachys glauca* (Cao et al. 2020), but not *C. sichuanensis*. In this study, we first sequenced and analyzed the complete chloroplast genome of *C. sichuanensis* genome-skimming sequencing to clarify the classification status of the species to promote the researches on the genus of *Chimonobambusa*.

Fresh and young leaf materials of *C. sichuanensis* were collected from the outdoor Bamboo Garden of Nanjing Forestry University, China (32°01′ N, 118°48′ E). The voucher specimen (JXAU-20201439) was deposited at the herbarium of the College of Forestry, Jiangxi Agricultural University, China. Illumina paired-end (PE) library was prepared and sequenced in the Nanjing Novogene Bio-technology Co., Ltd. (Nanjing, China). The complete chloroplast genome sequence was annotated using Geneious 9.0.5. The complete chloroplast genome sequence of *C*. *sichuanensis* was deposited in GenBank under the accession number of MT941921.

The complete genome sequence of *C. sichuanensis* was a circular DNA molecule of 139,594 bp in length, contains a small single-copy (SSC) region of 12,820 bp and a large single-copy (LSC) region of 83,196 bp, which were separated by a pair of inverted repeat (IR) regions of 21,789 bp. The overall GC content of the whole genome is 38.9%, and the corresponding values of the LSC, SSC, and IR regions are 37.0%, 33.2%, and 44.2%, respectively. The genome contained 140 genes, including 93 protein-coding genes, seven ribosomal RNA (rRNA) genes, and 40 transfer RNA (tRNA) genes.

To confirm the phylogenetic position of *C. sichuanensis*, a phylogenetic analysis was performed based on 24 complete chloroplast genomes as well as four species as outgroups, were downloaded from NCBI GenBank. The ML tree and Bayes tree were constructed by RAxML (Stamatakis 2014) and MrBayes 3.2.6 (Ronquist and Huelsenbeck 2003), respectively. The phylogenetic tree showed *C. sichuanensis* is clustered in the *Phyllostachys* clade, sister to *C. tumidissinoda* (Figure 1) with high bootstrap values. The internodes in the phylogenetic tree are short, indicating the probable recent rapid radiation during the evolution.

**Figure 1.**
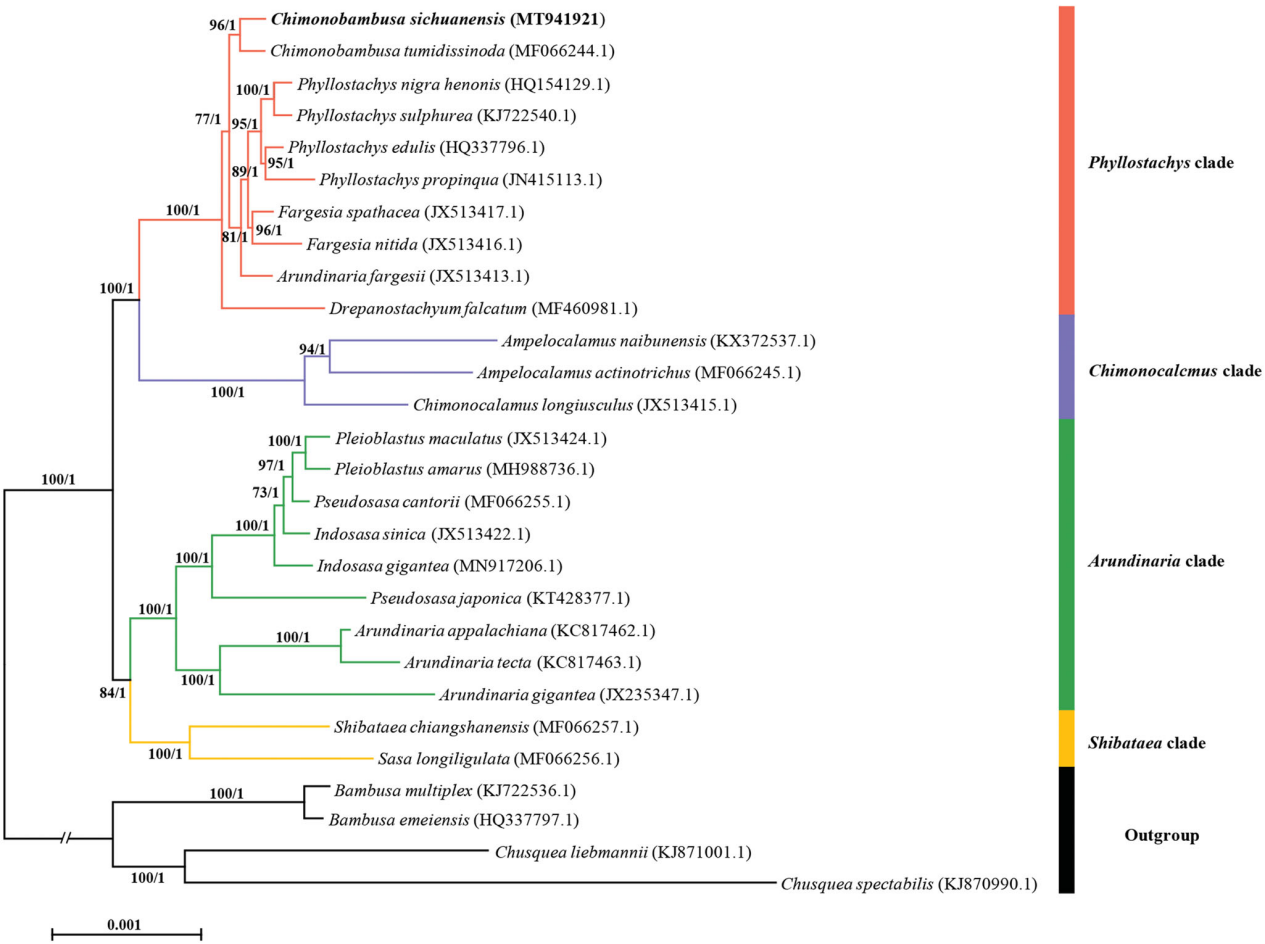
Maximum-likelihood tree based on complete chloroplast genomes from 28 bamboos showing the phylogenetic position of *C*. *sichuanensis*. Numbers associated with branches are ML bootstrap values, and Bayesian posterior probabilities, respectively.

## Data Availability

The genome sequence data that support the findings of this study are openly available in GenBank of NCBI at https://www.ncbi.nlm.nih.gov/ under the accession no. MT941921. The associated BioProject, SRA, and Bio-Sample numbers are PRJNA688799, SUB8802233, and SAMN17189334, respectively.
